# SeMet attenuates OTA-induced PCV2 replication promotion by inhibiting autophagy by activating the AKT/mTOR signaling pathway

**DOI:** 10.1186/s13567-018-0508-z

**Published:** 2018-02-13

**Authors:** Gang Qian, Dandan Liu, Junfa Hu, Fang Gan, Lili Hou, Nianhui Zhai, Xingxiang Chen, Kehe Huang

**Affiliations:** 1 College of Veterinary Medicine, Nanjing Agricultural University, Nanjing, 210095 Jiangsu Province China; 2 Institute of Nutritional and Metabolic Disorders in Domestic Animals and Fowls, Nanjing Agricultural University, Nanjing, 210095 Jiangsu Province China

## Abstract

Porcine circovirus type 2 (PCV2) is recognized as the causative agent of porcine circovirus-associated diseases. PCV2 replication could be promoted by low doses of ochratoxin A (OTA) as in our previous study and selenium has been shown to attenuate PCV2 replication. However, the underlying mechanism remains unclear. The aim of the study was to investigate the effects of selenomethionine (SeMet), the major component of organic selenium, on OTA-induced PCV2 replication promotion and its potential mechanism. The present study demonstrates that OTA could promote PCV2 replication as measured by cap protein expression, viral titer, viral DNA copies and the number of infected cells. In addition, OTA could activate autophagy as indicated by up-regulated light chain 3 (LC3)-II and autophagy-related protein 5 expressions and autophagosome formation. Further, OTA could down-regulate p-AKT and p-mTOR expressions and OTA-induced autophagy was inhibited when insulin was applied. SeMet at 2, 4 and 6 μM had significant inhibiting effects against OTA-induced PCV2 replication promotion. Furthermore, SeMet could attenuate OTA-induced autophagy and up-regulate OTA-induced p-AKT and p-mTOR expression inhibition. Rapamycin, an inhibitor of AKT/mTOR, could reverse the effects of SeMet on OTA-induced autophagy and the PCV2 replication promotion. In conclusion, SeMet could block OTA-induced PCV2 replication promotion by inhibiting autophagy by activating the AKT/mTOR pathway. Therefore, SeMet supplementation could be an effective prophylactic strategy against PCV2 infections and autophagy may be a potential marker to develop novel anti-PCV2 drugs.

## Introduction

Porcine circovirus type 2 (PCV2), a non-enveloped circular [1] single stranded DNA virus from the *Circoviridae* family [2], is the etiological agent of porcine circovirus–associated diseases (PCVAD), including post-weaning multi-systemic wasting syndrome [3], porcine respiratory disease complex [4], enteric disease [5], reproductive disease [6], which is arguably one of the most economically important diseases affecting the swine industry worldwide. However, not all pigs infected with PCV2 will develop PCVAD, other factors, such as animal management, coinfection and immunostimulation, have been reported to be associated with the disease [7]. Our recent findings suggested that both oxidative stress and ochratoxin A (OTA) enhance PCV2 replication [8, 9], which may partly explain the difference in morbidity and severity of PCVAD among PCV2-infected pig farms.

Autophagy is an evolutionarily conserved process that mediates the degradation of long-lived proteins and damaged organelles for recycling in response to diverse stress stimuli, including starvation, oxidative stress and viral infection [10–12]. During this process, sections of cytoplasm are sequestered within double-membrane vesicles (named autophagosomes) that eventually fuse with lysosomal compartments for bulk degradation [13, 14]. The kinase mammalian target of rapamycin (mTOR) is known to be a major modulator of autophagy [15]. AKT is an upstream molecular of kinase mTOR, and the activation of AKT could inhibit autophagy [16]. In recent years, interaction between autophagy and viral infection have been revealed in numerous studies. Normally, autophagy acts as a host antimicrobial defense mechanism against a variety of pathogens, including bacteria and viruses by delivering them to the lysosomal compartment [17, 18]. However, some viruses such as hepatitis C virus and dengue virus have developed strategies to utilize autophagy for their own benefit of replication [19, 20]. Previous studies suggested that PCV2 virus induces autophagy by suppressing the mTOR signaling pathway, and the inhibition of autophagy reduces the replication of PCV2 [21, 22].

Selenium is an essential trace element for humans and animals [23] which plays an important role in both the antioxidant defense system [24] and normal immune system [25] through its incorporation into selenoproteins such as glutathione peroxidases (GPxs), thioredoxin reductases (TrxRs), and iodothyronine deiodinases (DIOs) [26]. Selenium-deficiency has been reported to be linked to high occurrence, enhanced virulence or progression of some viral infections such as coxsackievirus, influenza, SARS coronavirus and HIV infections in humans and animals [27, 28]. In the meantime, selenium supplementation could serve as an appropriate adjuvant therapy to many viral infections including PCV2 [29, 30]. Our previous studies have shown that selenium supplementation could inhibit PCV2 replication through the regulation of selenoproteins in redox status [9, 31], indicating that selenium has a protective effect against PCV2 infection. However, whether selenium exerts its anti-PCV2 effect through autophagy remains unclear.

The present study was conducted to determine the effects of selenium on OTA-induced PCV2 replication promotion and its potential mechanism related to autophagy.

## Materials and methods

### Reagents and antibodies

Rapamycin (R0935), rabbit polyclonal anti-LC3B antibody (L7543) and horseradish peroxidase (HRP)-conjugated goat anti-rabbit or -mouse secondary antibodies were purchased from Sigma-Aldrich (St. Louis, USA). Rabbit polyclonal anti-ATG5 antibody (sc-33210) and mouse monoclonal anti-β-actin antibody (sc-47778) were purchased from Santa Cruz Biotechnology (Santa Cruz, USA). Rabbit anti-p-AKT (Ser473) antibody (4060) and anti-p-mTOR antibody (Ser2448) (5536) were purchased from Cell signaling Technology (Beverly, USA). Rabbit anti-AKT antibody (ab32505) and anti-mTOR antibody (ab2732) were purchased from Abcam (Cambridge, UK). 4′, 6-diamidino-2-phenylindole (DAPI) (C1005) and insulin (P3376) were purchased from Beyotime Biotechnology (Haimen, China).

### Cells, viruses and plasmids

PK-15 cells were provided by the China Institute of Veterinary Drug Control. The cells were cultured in Dulbecco’s modified Eagle’s medium (DMEM, Invitrogen, Carlsbad, USA) supplemented with 10% fetal bovine serum (FBS), 100 U/mL of penicillin, and 100 μg/mL of streptomycin at 37 °C in a 5% CO_2_ atmosphere.

The PCV2 strain (PCV2NJ2002) used in the experiment was originally isolated from a kidney tissue sample of a pig with naturally occurring PMWS. The PCV type was determined by sequencing (Invitrogen). PCV2 stocks were propagated on the PK-15 cell according to the procedures described before [31].

To construct pEGFP-LC3B, the LC3B gene was amplified from PK-15 cells with primers (LC3F: GAAGATCTGGGCTGAGGAGACACAAGAG; LC3R: CGGAATTCTCTCAGTTGGTAACATCCCTTT) that were designed based on the sequence of LC3B. The gene was then cloned into pEGFP-C1 to express LC3B fused with the EGFP protein at its N-terminus.

### Cell viability assay

Cell viability was determined by the MTT assay according to the manufacturer’s instructions (Sigma). Briefly, PK-15 cells were cultured in a 96-well plate at a density of 5 × 10^3^ cells/well for 24 h before the cells were treated with different concentrations of selenium for another 72 h. Then the reagent 3-(4,5-cimethylthiazol-2-yl)-2,5-diphenyl tetrazolium bromide (MTT) was added to the medium. After 4 h, the medium containing MTT was aspired and replaced by 150 μL DMSO for 30 min. Then the absorbance was measured using a multiplate reader at the wavelength of 550 nm. All tests were performed three times.

### LDH activity assay

LDH activity was determined using Pierce LDH Cytotoxicity Assay Kit (Thermo Fisher Scientific, Waltham, USA). Briefly, PK-15 cells were cultured in a 12-well plate at a density of 5 × 10^4^ cells/well with corresponding treatment. Then the medium was collected and centrifuged at 12 000 rpm for 10 min, and the supernatant was assayed according to the manufacturer’s instructions [32]. LDH activity was normalized to protein concentrations and the results are expressed as percentage of the control values. All tests were performed three times.

### Quantitative real-time PCR

PCV2 DNA copies in PK-15 cells were determined by quantitative real-time PCR as previously described [8]. In brief, DNA extractions were carried out using the TaKaRa DNA Mini kit (TaKaRa, Dalian, China). The purified DNA was used as a template for PCR amplification. A pair of PCV2-specific primers (forward primer 5′-TAGTATTCAAAGGGCACAG-3′, reverse primer 5′-AAGGCTACCACAGTCAG-3′) was designed to amplify a 117-bp fragment from the PCV2 ORF2 gene. Quantitative real-time PCR was performed using the ABI Prism Step One Plus detection system (Applied Biosystems, Foster city, USA). A recombinant pMD19 plasmid vector (TaKaRa) containing a PCV2 genome insert as a reference and a TaKaRa SYBR green real-time PCR kit was used to measure the amount of viral DNA.

### Indirect immunofluorescence assay (IFA)

PCV2 infected PK-15 cells were assayed by IFA as described previously [33]. PK-15 cells were fixed in 4% paraformaldehyde for 20 min then washed three times with PBS containing 0.1% Tween-20 (PBST). After fixation, the cells were perforated with 0.1% Triton X-100 and then incubated in PBST containing 1% bovine serum albumin (BSA) for 1 h at 37 °C to block nonspecific binding. Then, the cells were incubated with porcine anti-PCV2 antibody (UnivBiotech, Shanghai, China) for 1 h at 37 °C, and after three washes with PBST, FITC-conjugated rabbit anti-pig antibody (Sigma) was added and incubated for 1 h at 37 °C. After washing with PBST, the cells were examined under a fluorescence microscope. Cells positive for PCV2 viral antigens were counted in six fields of view.

### Quantification of virus titer

Total virus yield (intracellular and extracellular viruses) were determined by inoculating tenfold dilutions into confluent PK-15 cells in 96-well culture plates. After 72 h of incubation, the viral antigen was detected using indirect immunofluorescence assay (IFA) as described above. Virus titers were calculated using the Reed–Muench method and expressed as TCID_50_/mL [34].

### Western blotting analysis

PK-15 cells were collected with the cell lysis buffer containing protease inhibitor (Beyotime) on ice. Then the cell lysates were sonicated using a Snoics VCX105 sonicator and centrifuged at 12 000 rpm for 20 min at 4 °C. Protein concentration was determined by the BCA protein assay kit (Beyotime). Equal amounts of protein samples were diluted in 5 × SDS-PAGE loading buffer and heated at 95 °C for 5 min. The samples were separated on 12% SDS-PAGE gels and transferred to polyvinylidene fluoride (PVDF) membranes. After blocking for 1 h at RT in Tris-buffered saline (TBS) containing 5% nonfat milk powder and 0.1% Tween 20, the membranes were reacted with primary antibodies overnight at 4 °C. The membranes were washed and incubated in secondary antibody (polyclonal anti-rabbit–horse radish peroxidase from Sigma) at room temperature for 1 h. Blots were visualized according to the standard enhanced chemiluminescence system (Bio-Rad, Berkeley, USA). Quantification of protein blots was performed using the Image-Pro Plus 6.0 software (Media Cybernetics, Sarasota, USA), and images were acquired from an EU-88 image scanner (Seiko Epson Corporation, Suwa, Japan).

### Confocal immunofluorescence

Confocal fluorescence microscopy was used for analysis of LC3 expression after OTA treatment. Specifically, PK-15 cells grown on coverslips from 40 to 50% confluence were transfected with plasmid GFP-LC3 by an X-tremeGENE HP DNA transfection reagent (Roche, Nutley, USA) according to the manufacturer’s guidelines (Roche, USA). The localization of LC3 was visualized using a Nikon C1-si confocal fluorescence microscope (Nikon Instruments, Tokyo, Japan).

### Statistical analysis

Statistical analyses were performed using the SPSS (version 22.0). Data were analyzed using one-way analysis of variance (ANOVA) followed by least-significant difference test. Data are expressed as the mean ± standard error (SE). Differences were regarded as significant at *P* < 0.05.

## Results

### Cytotoxic effects of various concentrations of SeMet on PK-15 cells

**Figure 1:**
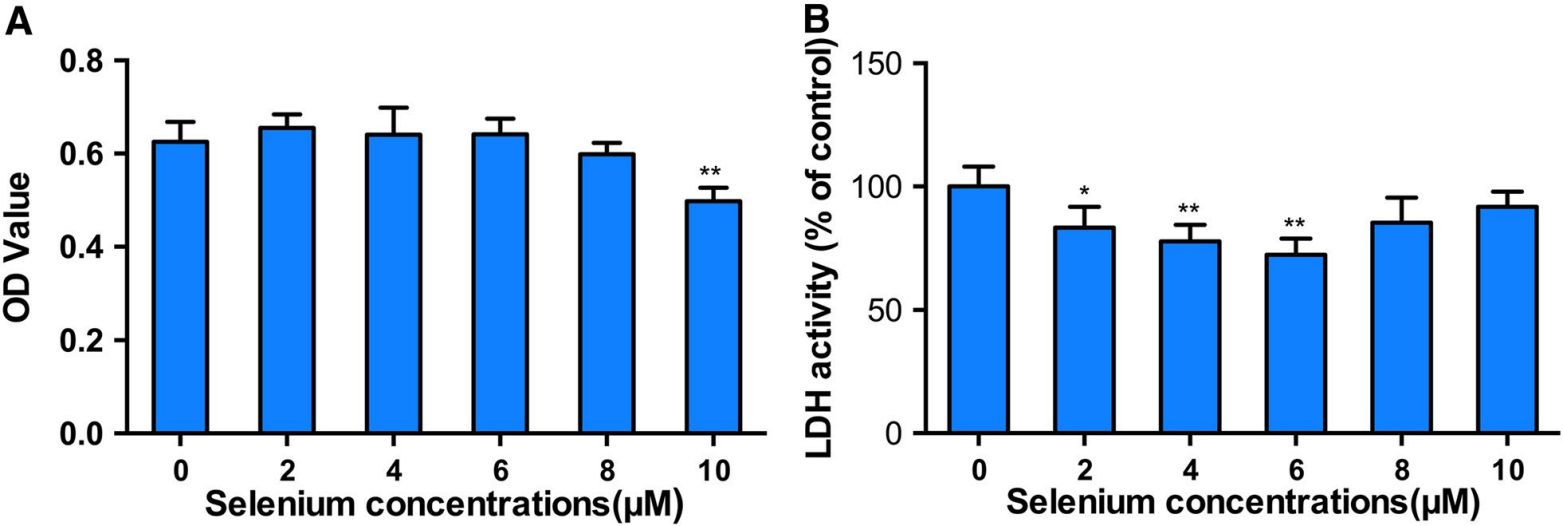
**Effects of selenium on cell viability and LDH activity in PK-15 cells.** PK-15 cells were seeded in 96-well plates at a density of 5 × 10^3^ cells/well and cultured with selenium at various concentrations for 72 h. Cell viability (**A**) and LDH activity (**B**) were determined as described in the materials and methods. Data are presented as mean ± SE of three independent experiments. Significance compared with control (0 μM Se), *P < 0.05 and **P < 0.01.

To assess the cytotoxic effect of selenium on PK-15 cells, SeMet at various concentrations were cultured with PK-15 cells for 72 h, then the cell viability and LDH activity were measured. As shown in Figure 1, over the range of concentrations used, the viability of PK-15 cells was not affected by SeMet at the concentrations of 2, 4 and 6 μM. However, PK-15 cell viability was reduced by SeMet (8 μM) (*P* > 0.05) and significantly reduced by SeMet (10 μM) (*P* < 0.01). In addition, SeMet treatment could significantly reduce LDH activity at concentrations of 2, 4 and 6 μM (*P* < 0.05). Therefore, SeMet at the concentrations of 2, 4 and 6 μM were selected for subsequent experiments.

### The inhibitory effect of SeMet on OTA-induced PCV2 replication promotion in PK-15 cells

**Figure 2:**
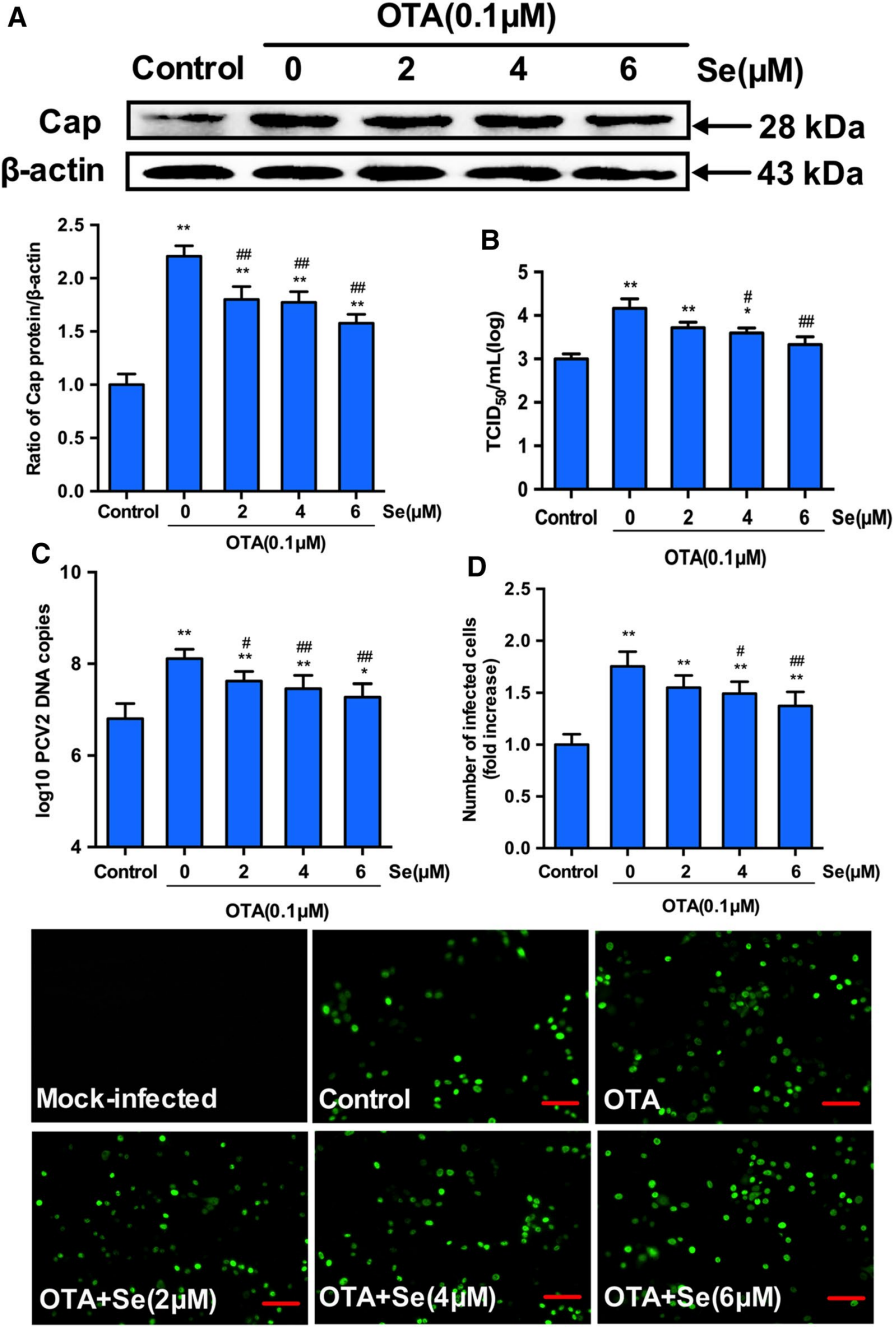
**Selenium blocks the PCV2 replication promoted by OTA.** PK-15 cells were cultured for 12 h with selenium at various concentrations and then treated with PCV2 in the presence or absence of 0.1 μM OTA for another 60 h. Cells were assayed for **A** PCV2 viral cap protein expression by Western blotting, **B** PCV2 viral DNA copies by Quantitative real-time PCR, **C** virus titers and **D** the number of infected cells (Scar bar: 100 μm) by IFA as described in Materials and methods. Data are presented as mean ± SE of three independent experiments. Significance compared with control, *P < 0.05 and **P < 0.01. Within the OTA-treated groups, significance compared with cells without selenium, ^#^*P* < 0.05 and ^##^*P* < 0.01.

To determine the effect of SeMet supplementation on OTA-induced PCV2 replication promotion, PK-15 cells were cultured with or without SeMet for 12 h, and then incubated with PCV2 in the presence or absence of OTA for another 60 h. We then measured the PCV2 viral cap protein levels, viral titers, PCV2 DNA copies and the number of infected cells. As shown in Figure 2, among all groups without SeMet treatment, 0.1 μM OTA significantly increased the cap protein levels (Figure 2A), viral titers (Figure 2B), PCV2 DNA copies (Figure 2C) and the number of PCV2-infected cells (Figure 2D), indicating 0.1 μM OTA could significantly promote PCV2 replication in PK-15 cells (*P* < 0.01). However, this promotion of PCV2 induced by OTA was significantly decreased when SeMet was added at concentrations of 2, 4 or 6 μM. And the inhibitory effect of SeMet was in a dose-dependent manner (Figure 2). These results indicate that SeMet has an inhibitory effect on OTA-induced PCV2 replication promotion.

### Effects of OTA treatment on autophagy in PCV2-infected PK-15 cells

**Figure 3:**
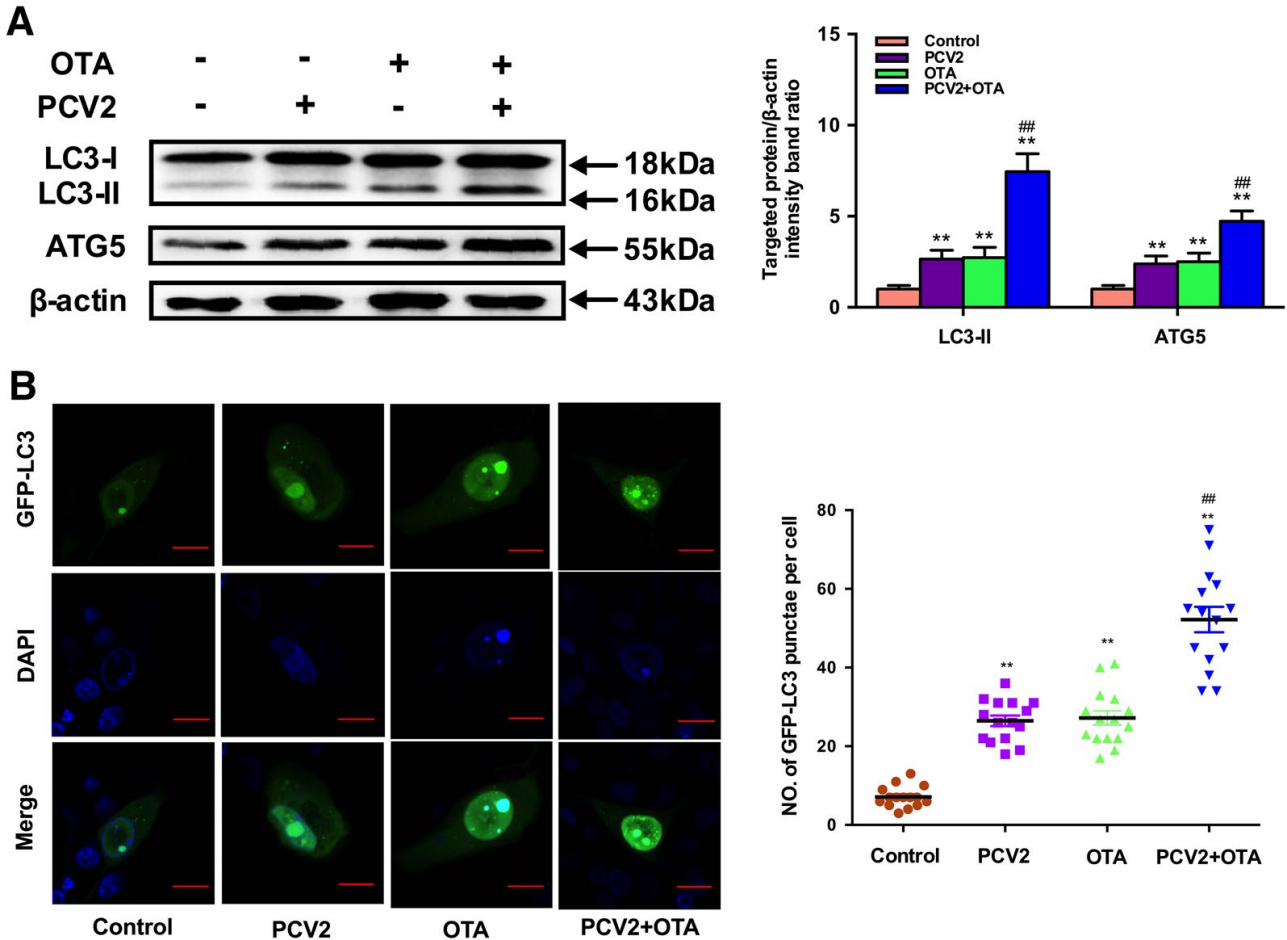
**Effects of OTA treatment on autophagy in PCV2-infected PK-15 cells.** **A** PK-15 cells were infected with or without PCV2 for 24 h then incubated in the presence or absence of 0.1 μM OTA for 48 h. After harvest, the expressions of LC3, ATG5 and β-actin were analyzed by Western blotting as described in Materials and methods. Data are presented as mean ± SE of three independent experiments. Significance compared with control, *P < 0.05 and **P < 0.01. Significance compared with PCV2, ^#^*P* < 0.05 and ^##^*P* < 0.01. **B** PK-15 cells were transfected with GFP-LC3 plasmid. After 12 h, PK-15 cells were infected with or without PCV2 for 24 h then incubated in the presence or absence of 0.1 μM OTA for 48 h and the fluorescence signals were visualized by confocal immunofluorescence microscopy (Scale bar: 10 μm). The average number of LC3 puncta in each cell was determined from at least 100 cells in each group. Data are presented as mean ± SE of three independent experiments. Significance compared with control, *P < 0.05 and **P < 0.01. Significance compared with PCV2, ^#^*P* < 0.05 and ^##^*P* < 0.01.

To determine the effects of OTA treatment on autophagy in PCV2-infected PK-15 cells, we first measured the levels of LC3-II and ATG5. PK-15 cells seeded at a density of 2 × 10^5^ cells/well in 6-well plates were treated with PCV2 at an MOI of 1 for 72 h or infected with PCV2 for 24 h then incubated with 0.1 μM OTA for an additional 48 h. The cells were then harvested and levels of LC3-II and ATG5 were determined by Western blotting. PCV2 infection or OTA treatment alone significantly increased LC3-II and ATG5 expressions when compared with the control group (*P* < 0.05) as shown in Figure 3. OTA treatment combined with PCV2 infection produced stronger increases in the levels of LC3-II and ATG5 than OTA treatment or PCV2 infection alone. The results indicate that OTA treatment induces autophagy in PCV2-infected PK-15 cells.

To further confirm whether autophagy was triggered by OTA treatment in PCV2-infected PK-15 cells, autophagosome formation in PK-15 cells incubated with PCV2 or/and OTA was investigated. PK-15 cells were transfected with green fluorescent protein-microtubule-associated protein 1 light-chain 3 (GFP-LC3), a specific marker of autophagosomes, infected with or without PCV2 for 24 h then incubated in the presence or absence of 0.1 μM OTA for 48 h, and then the fluorescence signals were visualized by confocal immunofluorescence microscopy. As shown in Figure 3B, significant increases in the number of GFP-LC3 puncta were observed in PCV2 or/and OTA-treated groups compared with the control group (*P* < 0.05). These findings further confirmed that OTA induces autophagy in PCV2-infected PK-15 cells.

### OTA treatment induces autophagy by suppressing the AKT/mTOR signaling pathway

**Figure 4:**
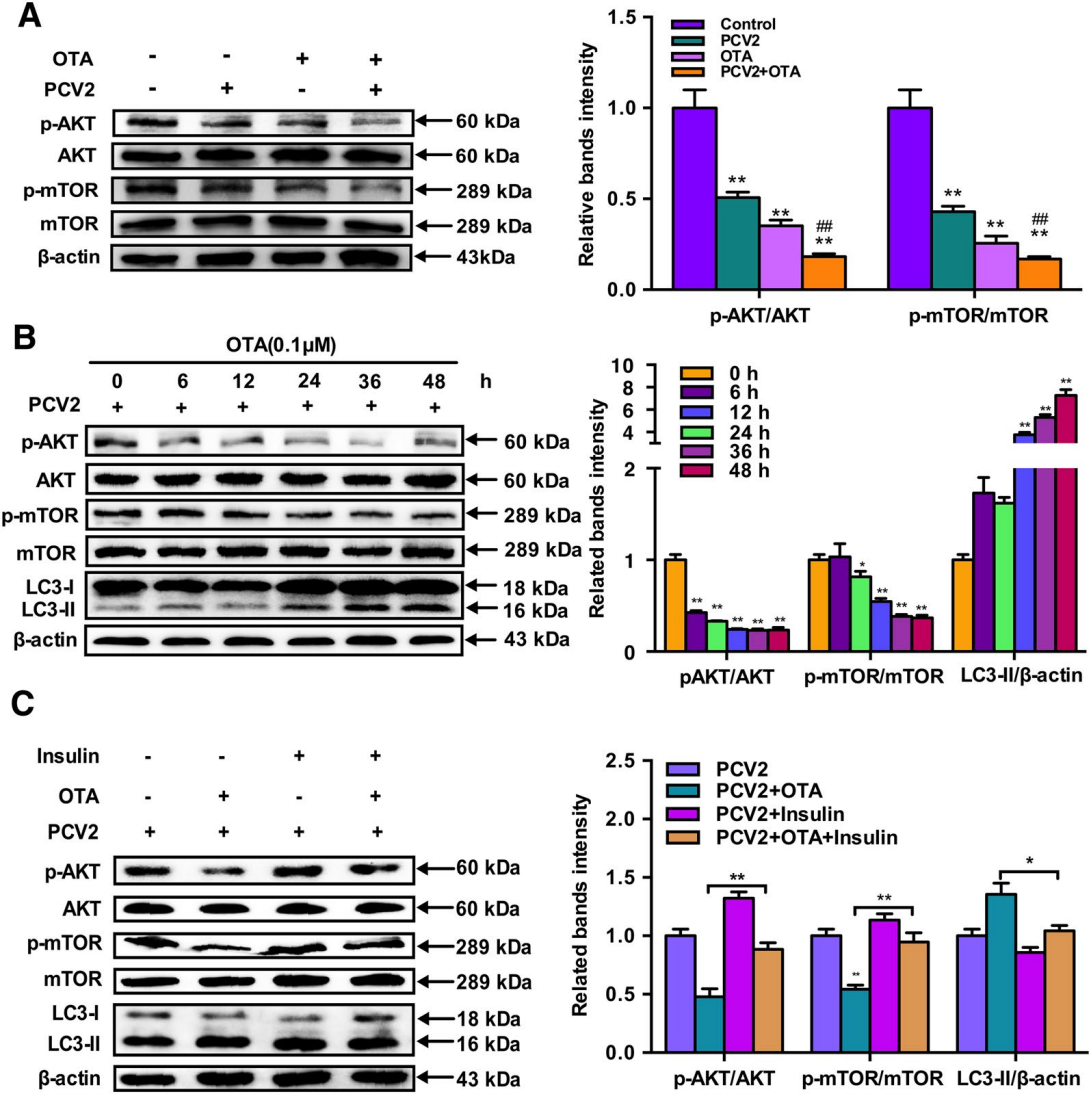
**OTA treatment induce autophagy by suppressing AKT/mTOR signaling pathway.** **A** PK-15 cells were infected with PCV2 for 24 h then incubated with 0.1 μM OTA for 48 h. Cells were harvested and the expressions of p-AKT, AKT, p-mTOR, mTOR, LC3 and β-actin were analyzed by Western blotting as described in Materials and methods. Data are presented as mean ± SE of three independent experiments. Significance compared with control, *P < 0.05 and **P < 0.01. Significance compared with PCV2, ^#^*P* < 0.05 and ^##^*P* < 0.01. **B** PK-15 cells were infected with PCV2 for 24 h then incubated with 0.1 μM OTA. Cells were harvested at the indicated time and the expressions of p-AKT, AKT, p-mTOR, mTOR, LC3 and β-actin were analyzed by Western blotting as described in Materials and methods. Data are presented as mean ± SE of three independent experiments. Significance compared with control, *P < 0.05 and **P < 0.01. **C** PK-15 cells were infected with PCV2 for 24 h then incubated with 0.1 μM OTA in the presence or absence of 100 μg/mL insulin for 48 h. After harvest, the expressions of p-AKT, AKT, p-mTOR, mTOR, LC3 and β-actin were analyzed by Western blotting as described in materials and methods. Data are presented as mean ± SE of three independent experiments. *P < 0.05 and **P < 0.01.

To further explore the underlying molecular mechanism of autophagy induced by OTA in PK-15 cells, AKT/mTOR signaling pathway, one of the major regulators of autophagy, was investigated [15]. PK-15 cells were infected with or without PCV2 for 24 h then incubated with 0.1 μM OTA for 48 h. Cells were harvested and expressions of p-AKT, AKT, p-mTOR and mTOR were analyzed by Western blotting. As shown in Figure 4A, phosphorylated AKT and mTOR were significantly down-regulated after the treatment of OTA (*P* < 0.05). To further determine the changes of AKT/mTOR pathway following the treatment of OTA in PCV2-infected PK-15 cells, PK-15 cells were infected with PCV2 for 24 h, then incubated with 0.1 μM OTA. Cells were harvested at the indicated times and phosphorylated AKT and mTOR were persistently down-regulated after the treatment of OTA. In the meantime, the ratio of LC3-II to β-actin was significantly increased in a time-dependent manner when compared with the control group (Figure 4B). These results indicate that AKT/mTOR signaling pathway may participate in the autophagy induced by OTA.

To further confirm the role of the AKT/mTOR pathway in OTA-induced autophagy, the activator of AKT/mTOR insulin was applied. As shown in Figure 4C, following the treatment of insulin, the decreases in phosphorylated AKT and mTOR were restored in OTA-treated or mock-treated PCV2-infected PK-15 cells, which led to a reduced ratio of LC3-II to β-actin, which indicating the activation of AKT/mTOR pathway suppressed OTA-induced autophagy. Taken together, our results confirm that OTA treatment induced autophagy by suppressing the AKT/mTOR signaling pathway.

### SeMet supplementation attenuates OTA-induced autophagy and activates AKT/mTOR signaling pathway in PCV2-infected PK-15 cells

**Figure 5:**
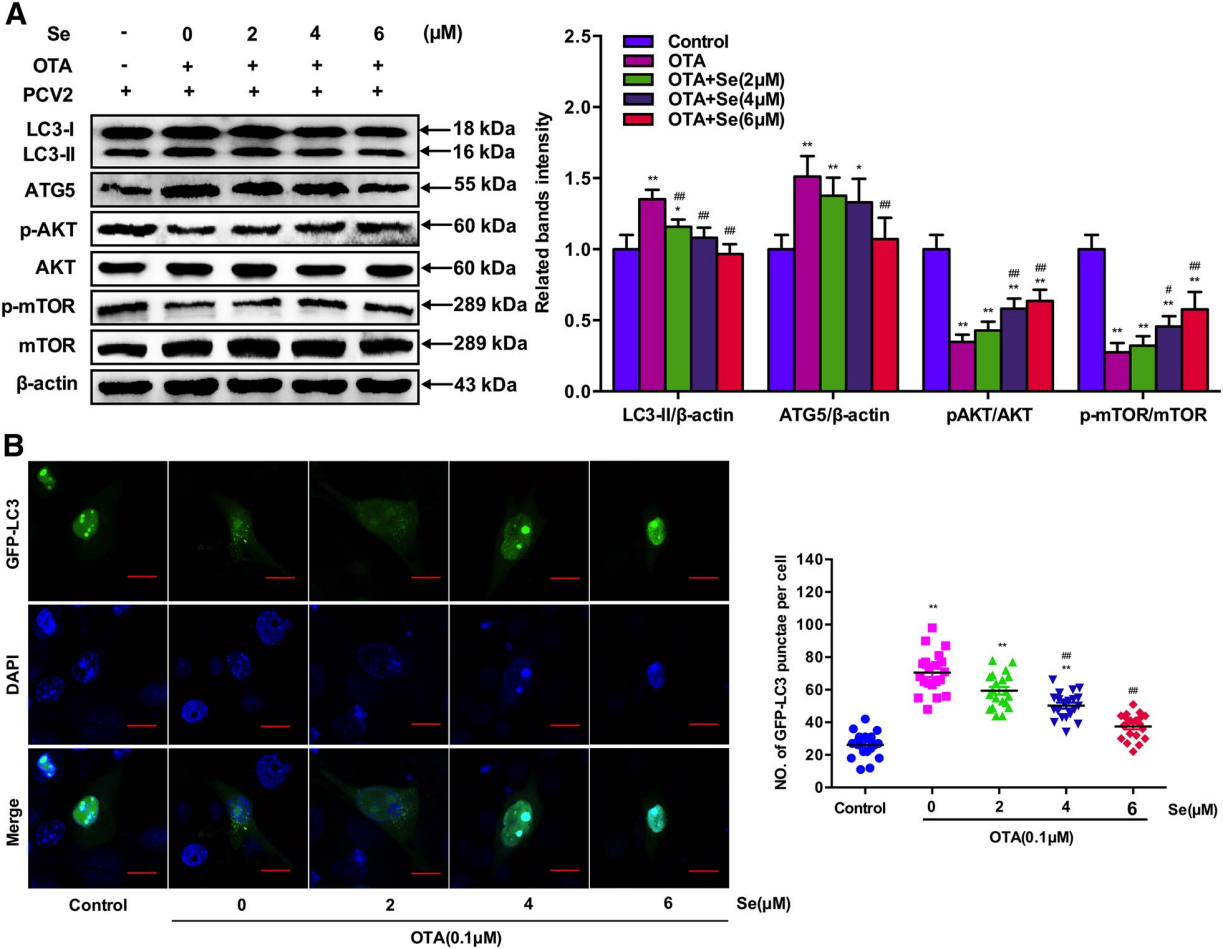
**Effects of selenium supplementation on OTA-induced autophagy in PCV2-infected PK-15 cells.** **A** PK-15 cells were cultured with or without selenium for 12 h then incubated with PCV2 and 0.1 μM OTA for another 60 h. After harvest, the expressions of LC3, ATG5, p-AKT, AKT, p-mTOR, mTOR and β-actin were analyzed by Western blotting as described in Materials and methods. Data are presented as mean ± SE of three independent experiments. Significance compared with control, *P < 0.05 and **P < 0.01. Significance compared with OTA, ^#^*P* < 0.05 and ^##^*P* < 0.01. **B** PK-15 cells were transfected with GFP-LC3 plasmid. Then PK-15 cells were cultured with or without selenium for 12 h and incubated with PCV2 and 0.1 μM OTA for another 60 h and the fluorescence signals were visualized by confocal immunofluorescence microscopy (Scale bar: 10 μm). The average number of LC3 puncta in each cell was determined from at least 100 cells in each group. Data are presented as mean ± SE of three independent experiments. Significance compared with control, *P < 0.05 and **P < 0.01. Significance compared with PCV2, ^#^*P* < 0.05 and ^##^*P* < 0.01.

PK-15 cells were seeded in 6-well plates at a density of 2 × 10^5^ cells/well with or without SeMet for 12 h. After incubation with PCV2 and 0.1 μM OTA for another 60 h, the levels of LC3-II and ATG5 were determined by Western blotting. As shown in Figure 5A, 0.1 μM OTA treatment led to significant increases in LC3-II and ATG5 levels when compared to the control group and these increases were smaller when 2 or 4 μM SeMet was added. Furthermore, this increase in LC3-II and ATG5 levels induced by OTA treatment were blocked when SeMet concentration was increased to 6 μM.

Meantime, the above results were further confirmed by the detection of autophagosome formation. GFP-LC3 transfected PK-15 cells were cultured with or without SeMet for 12 h and then incubated with PCV2 and 0.1 μM OTA for another 60 h before the fluorescence signals were visualized by confocal immunofluorescence microscopy. As shown in Figure 5B, significant increases in the GFP-LC3 puncta were observed in OTA-treated cells compared with the control group. However, these increases of GFP-LC3 puncta were smaller when 2 or 4 μM SeMet was added. Furthermore, these increases of GFP-LC3 puncta induced by OTA treatment were blocked when SeMet concentration was increased to 6 μM. These results suggest that SeMet supplementation may attenuate OTA-induced autophagy in PCV2-infected PK-15 cells.

Since OTA induced autophagy by inactivating the AKT/mTOR signaling pathway in PCV2-infected PK-15 cells, we wondered whether SeMet attenuated OTA-induced autophagy through the AKT/mTOR pathway. PK-15 cells were seeded in 6-well plates at a density of 2 × 10^5^ cells/well with or without SeMet for 12 h. After incubation with PCV2 and 0.1 μM OTA for another 60 h, the levels of p-AKT, AKT, p-mTOR and mTOR were determined. As shown in Figure 5A, p-AKT and p-mTOR levels were significantly down-regulated by OTA treatment in PCV2-infected cells. However, these down-regulations of p-AKT and p-mTOR levels were attenuated by SeMet supplementation in a dose-dependent manner. All these results indicate that SeMet could attenuate OTA-induced autophagy by activating the AKT/mTOR signaling pathway in PK-15 cells.

### Inactivation of the AKT/mTOR pathway abolishes the inhibitory effect of SeMet on OTA-induced autophagy and PCV2 replication promotion

**Figure 6:**
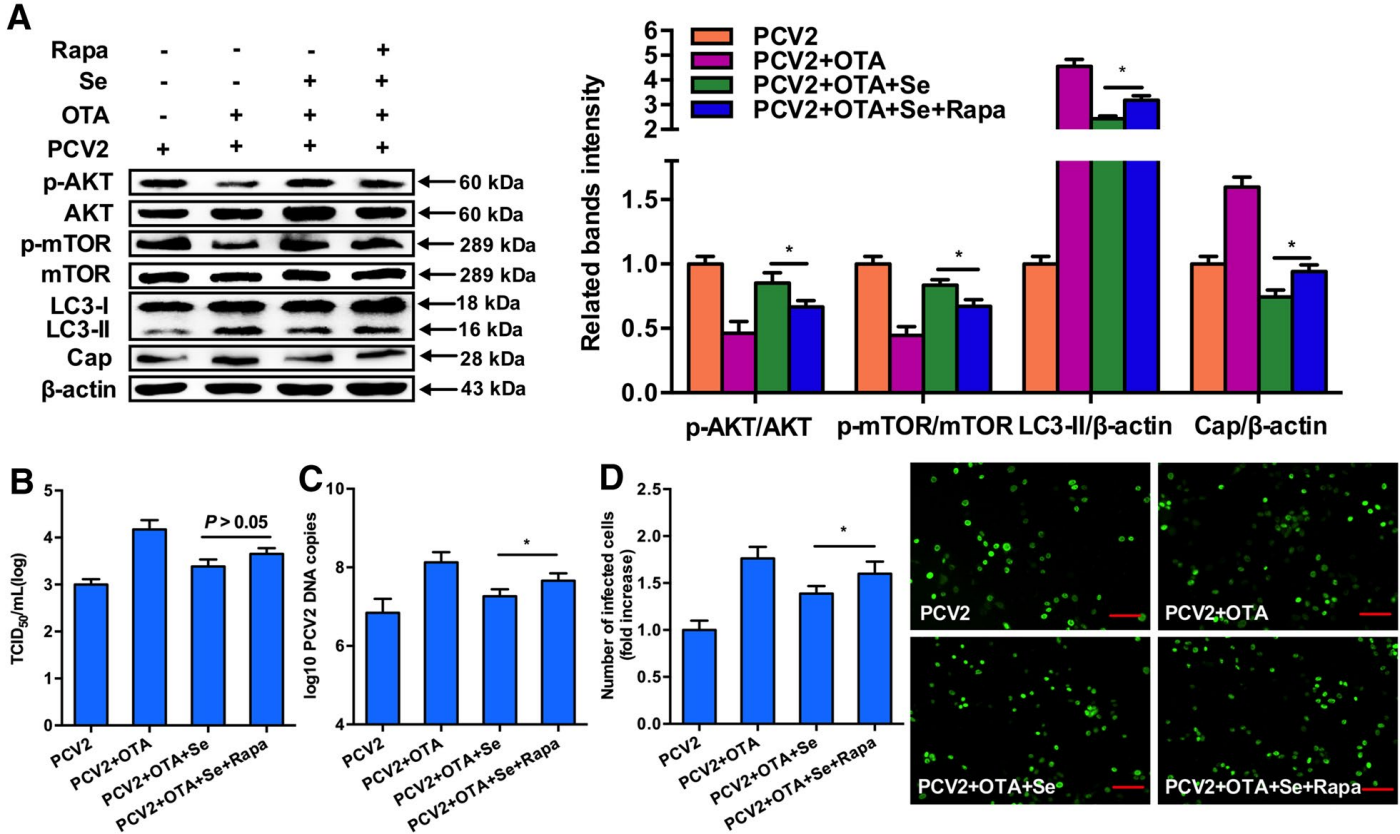
**Inactivation of AKT/mTOR signaling pathway abolishes the inhibitory effect of selenium on OTA-induced autophagy and PCV2 replication promotion.** PK-15 cells were pretreated with rapamycin and cultured with or without selenium for 12 h before incubation with PCV2 and 0.1 μM OTA for another 60 h. After harvest, **A** expressions of LC3, Cap, p-AKT, AKT, p-mTOR, mTOR and β-actin were analyzed by Western blotting. **B** Virus titers by IFA. **C** PCV2 viral DNA copies by Quantitative real-time PCR. **D** the number of infected cells (Scar bar: 100 μm) by IFA as described in materials and methods. Data are presented as mean ± SE of three independent experiments. *P < 0.05, **P < 0.01.

To further determine the functional significance of AKT/mTOR activation, PK-15 cells were pretreated with rapamycin, a well-known inhibitor of mTOR, and then cultured with or without SeMet for 12 h before incubation with PCV2 and 0.1 μM OTA for another 60 h. As shown in Figure 6A, treatment of rapamycin significantly suppressed SeMet-induced activation of p-mTOR and p-AKT (*P* < 0.05). In the meantime, treatment of rapamycin reversed SeMet-induced down-regulation of LC3 expression. The activation of the mTOR pathway partly abolished the inhibitory effect of SeMet on OTA-induced PCV2 replication promotion as demonstrated by the elevated cap expression levels (Figure 6A), viral titers (Figure 6B), PCV2 DNA copies (Figure 6C) and the number of infected cells (Figure 6D). The results suggest that inactivation of the AKT/mTOR pathway abolishes the inhibitory effect of SeMet on OTA-induced autophagy and PCV2 replication promotion.

## Discussion

PCVAD is now an emerging viral disease that poses great threats to the swine industry. Despite much progress in the PCV2vaccine, vaccine efficacy remains uncertain due to variations in concentration of antigen, adjuvant type and the administration doses [35]. Nutrition supplementation in trace elements is suggested to be an effective prophylactic protection against viral infections [36]. Selenium is an essential micronutrient for both humans and animals [37]. Selenium deficiency has been reported to be related to enhanced virulence and severity of some viruses including HIV and Coxsackie virus [38, 39], whereas selenium supplementation could improve the immune response to protect against viral infections. In HIV infection, selenium supplementation increased CD4^+^ T cells and suppressed the progression of viral loads [28]. And selenium supplementation could protect mice infected with H1N1 as well [40]. In this study, we determined the anti-viral activity of selenium against OTA-induced PCV2 replication promotion in PK-15 cells, and our results show that OTA promoted PCV2 replication by inducing autophagy through the AKT/mTOR pathway and selenium supplementation inhibited OTA-induced PCV2 replication promotion in a dose-dependent manner. Furthermore, we show that selenium supplementation exerts its protective effects by inhibiting autophagy through the activation of the AKT/mTOR signaling pathway.

Although PCV2 is recognized as the etiological agent of PCVAD, other infectious or non-infectious factors are required for the development of the disease [41]. Previous studies from our lab have demonstrated that low doses of OTA could promote PCV2 replication in vitro and in vivo through the regulation of oxidative stress and autophagy [33, 42], indicating that OTA may be an important trigger of PCV2 replication. In addition, our results further confirmed that 0.1 μM OTA could significantly promote PCV2 replication in PK-15 cells (Figure 2). In this paper, we used SeMet, an organic Se source as the selenium supplementation due to its lower toxicity compared to sodium selenite [31, 43]. And the SeMet concentration we selected in the experiment had no toxic effect on PK-15 cells (Figure 1). Our results show that SeMet supplementation was effective in protecting against OTA-induced PCV2 replication promotion, and in a dose-dependent manner (Figure 2). But the underlying related mechanisms need to be further investigated.

The autophagy pathway was initially identified as a process caused by cellular starvation, and is now known to play essential roles in numerous physiological and pathological processes [44]. Many viruses have been shown to affect this cellular pathway. Normally, autophagy acts as an innate immune response against viral infections [45]. Meantime, some viruses could utilize the autophagy machinery to enhance their own replication and survival [17, 46]. Studies have shown that autophagy was required for the replication of PCV2 [21, 22, 47], and our previous study showed that OTA-promoted PCV2 replication was mediated by autophagy [42]. In addition, our present results further confirmed that 0.1 μM OTA could induce autophagy in PCV2-infected PK-15 cells as demonstrated by up-regulated LC3-II and ATG5 expressions and autophagosome formation (Figure 3).

AKT/mTOR signaling pathway is a well-known regulator of autophagy, and the inhibition of the AKT/mTOR pathway could activate autophagy [48]. Recent studies showed that PCV2 could induce autophagy by inhibiting the AKT/mTOR pathway as well [21]. Therefore, we wonder whether OTA induced autophagy by inactivating the AKT/mTOR pathway. We observed that the levels of p-AKT and p-mTOR were down-regulated following the treatment of OTA and in a time-dependent manner (Figures 4A and B). To further determine the role of the AKT/mTOR pathway in OTA induced autophagy, we applied insulin, an activator of AKT/mTOR, and the results show that the OTA-induced down-regulation in AKT phosphorylation and mTOR expressions were restored after insulin treatment (Figure 4B), indicating OTA induced autophagy by inactivating the AKT/mTOR signaling pathway.

Normally, selenium exerts its biological effects when it is incorporated into selenoproteins, and most selenoproteins function as antioxidant enzymes. Our previous studies demonstrated that selenium could inhibit PCV2 replication mainly through the function of selenoproteins including GPx1 and selenoprotein S [9, 31]. Previous studies also showed that inhibition of autophagy could suppress viral replication, including PCV2 [22]. So, we wondered whether autophagy was involved in the mechanism underlying the anti-PCV2 effect of selenium. In the present study, we first demonstrate that SeMet supplementation prevented the activation of autophagy induced by OTA, as reflected by decreased levels of LC3-II, ATG5 and the number of GFP-LC3 spots (Figure 5). Our results show that OTA induced autophagy by inactivating the AKT/mTOR signaling pathway in PCV2-infected PK-15 cells (Figure 4). In addition, several studies have indicated that selenium could activate the PI3 K-AKT pathway [49], and studies showed that mTOR is directly linked to AKT, and the PI3 K-AKT-mTOR signaling pathway plays an important role in autophagy [48]. Thus, we speculate that the inhibiting effect of selenium on autophagy is due to its activation of the AKT/mTOR signaling pathway. In addition, our results further show that the levels of p-AKT and p-mTOR were restored after the treatment of SeMet in a dose-dependent manner, indicating that the AKT/mTOR signaling pathway was activated after the treatment of SeMet.

To further determine whether SeMet exerts its effects through the above mechanisms, we detected the effect of SeMet on OTA-induced autophagy and PCV2 replication promotion in the presence of rapamycin, a well-known inhibitor of mTOR and stimulator of autophagy [15]. And the treatment of rapamycin could increase the ratio of LC3-II/β-actin, as well as PCV2 replication, which partly attenuated the anti-autophagy and anti-PCV2 effects of SeMet. These observations indicate that SeMet might block OTA-induced PCV2 replication promotion by inhibiting autophagy through the activation of the AKT/mTOR signaling pathway.

In conclusion, our results show that the protective effect of selenium against OTA-induced PCV2 replication promotion was related to its inhibition of autophagy by activating the AKT/mTOR signaling pathway. Our findings indicate that selenium supplementation could be an effective method against PCV2 infection, and the regulation of autophagy will contribute to potential antiviral drug development.

## Abbreviations

PCV2: porcine circovirus type 2
OTA: ochratoxin a
SeMet: selenomethionine
mTOR: mammalian target of rapamycin
PCVAD: porcine circovirus–associated diseases
GPxs: glutathione peroxidases
TrxRs: thioredoxin reductases
DIOs: iodothyronine deiodinases
HRP: horseradish peroxidase
DMEM: Dulbecco’s modified Eagle’s medium
FBS: fetal bovine serum
IFA: indirect immunofluorescence assay
PVDF: polyvinylidene fluoride
TBS: tris-buffered saline
ANOVA: one-way analysis of variance
GFP-LC3: green fluorescent protein-microtubule-associated protein 1 light-chain 3
